# LongPolyASE: an end-to-end framework for allele-specific gene and isoform analysis in polyploids using long-read RNA-seq

**DOI:** 10.1186/s13007-026-01569-8

**Published:** 2026-07-21

**Authors:** Nadja Nolte, Kristina Gruden, Marko Petek

**Affiliations:** 1 Department of Biotechnology and Systems Biology, National Institute of Biology, Večna Pot 121, 1000 Ljubljana, Slovenia; 2 Jožef Stefan International Postgraduate School, Jamova Cesta 39, 1000 Ljubljana, Slovenia

**Keywords:** Allele-specific expression analysis, Polyploids, Long-read RNA-seq, Isoform expression

## Abstract

**Background:**

Allele-specific expression analysis can reveal *cis*-regulatory differences (e.g., promoter variants, epigenetic changes) that cause imbalanced gene expression between haplotypes. Haplotype-resolved reference genomes and long-read RNA sequencing enable allele-specific expression analysis at gene and isoform-levels. However, existing tools are largely restricted to short-read RNA sequencing data and diploid organisms.

**Results:**

We developed LongPolyASE, an end-to-end computational framework for allele-specific gene and isoform expression analysis in diploid and polyploid organisms using long-read RNA sequencing, consisting of three components: Syntelogfinder, for identifying syntenic gene relationships and annotation inconsistencies; longrnaseq, for novel isoform discovery and haplotype-level quantification; and PolyASE, for statistical testing and visualization of allelic imbalance and isoform usage. We applied LongPolyASE to diploid rice, autotetraploid potato, allotetraploid rapeseed, and allooctoploid strawberry using Oxford Nanopore and PacBio long-read RNA-seq. The framework enabled identification of *cis*-regulatory variation, tissue-specific *trans*-regulatory effects, differential isoform usage, and haplotype-specific splicing differences. In addition, it facilitated the discovery of novel transcripts and genes with potential functional relevance in plant development.

**Conclusions:**

LongPolyASE addresses a key methodological gap by enabling allele-specific expression analysis in polyploid organisms using long-read RNA sequencing. By combining haplotype-aware quantification with isoform-level resolution in a reproducible workflow, the framework provides a practical tool for plant researchers working with complex genomes. Its application to crop species highlights its potential to support the identification of regulatory variation and candidate targets for plant breeding.

**Supplementary Information:**

The online version contains supplementary material available at https://doi.org/10.1186/s13007-026-01569-8.

## Background

Haplotype-resolved (phased) reference genomes reconstruct the distinct chromosomal copies (haplotypes) present in an organism separately rather than collapsing them into a single consensus. These genome assemblies are becoming available for a wide range of organisms, from personal diploid genomes of humans [8] to haplotype-resolved genomes of polyploid plant cultivars such as hexaploid wheat [28], allooctoploid strawberry [22], allotetraploid rapeseed [44], and autotetraploid potato [20, 48], among others. These phased reference genomes simplify expression analysis on haplotype-level and reduce mapping bias in allele-specific expression (ASE) analysis [12].

Long-read RNA sequencing (RNA-seq) of full-length transcripts commercialized by Oxford Nanopore Technologies (ONT) and PacBio offers several key advantages over short-read RNA-seq: discovery of novel isoforms [39], improvement of isoform quantification [6], and unique assignment of transcripts from highly similar loci to their origin based on single nucleotide variations. Combined with phased reference genomes, long-read RNA-seq enables straightforward quantification of allele-specific expression at both gene and isoform-levels.

Allele-specific expression analysis can identify genes with imbalanced expression between haplotypes, revealing potential *cis*-regulatory differences, such as promoter variations or epigenetic modifications, that inhibit or increase gene expression from specific alleles. When comparing allelic expression across different conditions, e.g., developmental stages or environmental conditions, ASE analysis can identify *trans-*regulatory factors that differentially modulate haplotype expression in a condition-dependent manner, for instance condition-specific transcription factors [38].

However, several challenges limit the application of phased assemblies and long-read RNA-seq for allele-specific gene and isoform expression analysis in polyploids. The majority of tools and pipelines for allele-specific expression analysis are developed for short-read RNA-seq and diploid organisms [9, 37]. Current tools for long-read allele-specific gene and isoform analysis are phasing reads into maternal and paternal haplotype based on one haplotype reference genome sequences e.g. FLAIR2 [49], isoLASER [43], longcallR [25] or assigning reads based on alignments to phased genome sequences of diploid model organisms e.g. LORALS [19]. These tools lack options for analysis of more than two alleles and are therefore not applicable for polyploid organisms with phased assemblies. While few tools with polyploid support exist e.g. BYASE [15], they do not utilize phased references or long-read RNA-seq.

Phased reference genomes allow allele quantification without reference bias, but there are some limitations of their use. In phased reference assemblies, gene annotations are typically generated independently for each haplotype, resulting in non-unified gene identifiers and gene models across haplotypes. Therefore, syntelogs [26], orthologous or paralogous genes that maintain their relative chromosomal position across haplotypes, must be identified before comparative expression analysis. Additionally, inconsistent gene annotation between haplotypes, commonly arising from length differences in the annotation of untranslated regions (UTR), can lead to biased haplotype-level quantification [40].

**Fig. 1:**
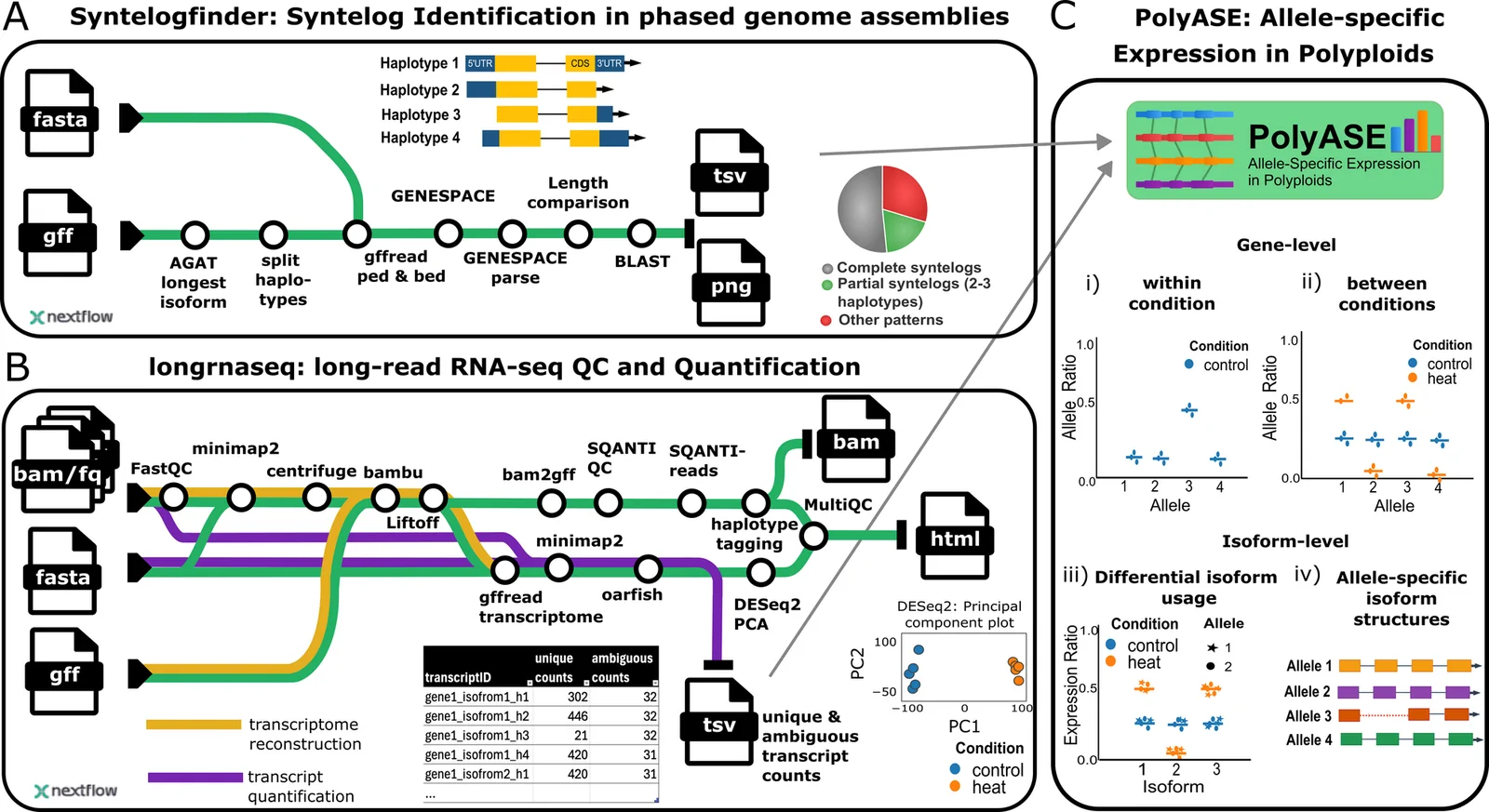
Overview of the allele-specific expression analysis framework for polyploid organisms using long-read RNA-seq. The framework consists of three integrated components: **A** Syntelogfinder identifies syntelogs across phased haplotypes using GENESPACE. Genes are categorized based on their copy number and syntenic relationships across haplotypes. Single-copy syntelogs, which are present on each haplotype with no gene duplications within any haplotype, are selected for allelic analysis. Steps in the pipeline are connected by a green line. **B** longrnaseq processes Oxford Nanopore Technologies (ONT) and PacBio long-read RNA data for discovery of novel isoforms and genes (yellow) and isoform-level quantification (purple). First, the RNA-seq reads are aligned to a phased reference genome with minimap2, novel transcripts are discovered with Bambu, novel annotations are transferred across haplotypes with Liftoff, and transcripts are quantified with Oarfish. Further, quality control is performed with SQANTI3, SQANTI-reads, and Centrifuge, with comprehensive MultiQC reporting. Lastly, haplotype-tagged alignments enable visualization of allele-specific gene and isoform expression in genome browsers. **C** PolyASE performs statistical analysis and visualization of allele-specific expression patterns, including: (i) identification of genes with allelic imbalance within conditions (*cis*-regulatory variation), (ii) detection of condition-dependent changes in allelic ratios (*trans-*regulatory effects), (iii) differential isoform usage testing between conditions, and (iv) identification of structural differences in major isoforms between haplotypes

To address the gap in tools and the challenges of phased reference genomes for the analysis of haplotype-specific expression using long-read RNA-seq in polyploids, we developed an end-to-end framework for allele-specific gene and isoform expression analysis using long-read RNA-seq in diploid and polyploid organisms (Fig. 1). The framework consists of three components: (1) Syntelogfinder, a Nextflow [13, 32] pipeline that groups genes by syntenic relationships and identifies length differences; (2) longrnaseq, a Nextflow pipeline for novel isoform discovery, haplotype-level quantification, and quality control; and (3) PolyASE, a Python package for statistical testing and visualization of allelic imbalance, condition-dependent expression differences, differential isoform usage, and variation in major-expressed isoforms between haplotypes. We tested the framework on diploid rice, autotetraploid potato, allotetraploid rapeseed and allooctoploid strawberry, using ONT and PacBio long-read RNA-seq, identifying *cis*-regulatory variation, tissue-specific trans-regulatory effects, differential isoform expression of a newly identified gene linked to rice tillering regulation, and haplotype-specific splicing differences in potato.

## Methods and implementation

### Pipeline overview

We developed three integrated tools for analyzing allele-specific and isoform expression in polyploid genomes (Fig. 1): Syntelogfinder for identifying syntenic gene relationships, longrnaseq for processing long-read RNA-seq data, and PolyASE for downstream analysis and statistical testing.

### Identification of syntenic genes

The Syntelogfinder pipeline (Fig. 1A) enables syntelog identification from phased reference genome sequences when gene annotations and a phased assembly are available. The pipeline splits annotations by haplotype, extracts the longest isoform per gene with agat_sp_keep_longest_isoform.pl v1.4.0 [27], obtains protein sequences of protein-coding genes, and runs GENESPACE v1.3.1 [36], which executes OrthoFinder2 [16] and MCScanX [51]. GENESPACE was configured with onlySameChrs = True to restrict syntelog identification to the same chromosome and ploidy = 1 as genomes were separated into haplotypes, with one haplotype randomly designated as "refGenome". The query_pangenes function identified gene groups with orthologous, paralogous, and syntenic relationships. Pangene output was parsed using a custom script (parse_genespace_pangenes.py) to assign genes to groups based on haplotype presence and synteny. Single-copy syntelogs, i.e. those present on each haplotype with no gene duplications within any haplotype, were analyzed for sequence length differences and transcript similarity via BLASTn v2.16.0 [2].

### long-read RNA-seq processing and quantification

The longrnaseq pipeline (Fig. 1B) processes PacBio and ONT long-read RNA-seq data through two subworkflows: novel transcript discovery and quantification. Raw reads were quality-checked with FastQC v0.12.1 and aligned to phased reference genome sequences using minimap2 v2.29-r1283 [33, 34]. ONT cDNA reads were aligned to the genome with parameters "-ax splice -N 200 –secondary = yes -G 10000 –secondary-seq", and to the transcriptome with "–eqx -N 200 -ax map-ont". PacBio reads were aligned with "-ax splice:hq -uf -N 200 –secondary = yes -G 10000 –secondary-seq" (genome) and "–eqx -N 200 -ax map-hifi" (transcriptome). For large genomes, the "-I 16 g" option was added.

Novel transcripts and genes were identified from genome alignments using Bambu v3.0.8 [7] with default parameters. To mitigate haplotype-specific annotation bias, Bambu gene models were transferred between haplotypes using Liftoff [46] with parameters "-infer_genes -a 0.7 -s 0.7 -copies -sc 0.7 -overlap 1.0". Liftoff and original annotations were merged with gffcompare v0.12.7 [42] and identifiers were corrected with a custom script. Transcript quality control was performed with SQANTI3 v5.5.1 [41] with parameters "-min_ref_len 0 –skipORF" and SQANTI-reads v1.0 [30] with parameters "–force_id_ignore –pca_tables".

Transcripts were extracted with gffread v0.12.7, reads were then aligned to transcripts with minimap2, and quantified using oarfish v0.9.0 [54] with parameters "–filter-group no-filters –write-assignment-probs –score-threshold 1.0". Unaligned reads were screened for contamination using Centrifuge v1.0.4 (database build date: 03.03.2018). For visualization, aligned reads were subsampled using samtools view v1.21 [10] and assigned a haplotype ('HP') tag based on the location of the primary alignment. This enables visualization of allele-specific expression and isoform structures in genome browsers such as JBrowse2 [14]. Quality control reports were generated with MultiQC v1.25.1 [18]. The pipeline outputs transcript-level counts for uniquely and ambiguously mapping reads at the haplotype-level, is modular and containerized following nf-core principles [13, 17, 32], was tested on a SLURM-managed HPC cluster, and handles large genome sequences including phased wheat.

### Downstream analysis of allelic expression

All downstream processing was implemented in PolyASE v1.3.6 (Fig. 1C), a Python package for inspection of allelic counts and testing for imbalanced expression of syntenic genes, as well as isoform usage and structure. The necessary input files are transcript counts and grouping of genes based on syntenic relationships, which are generated by the two described Nextflow pipelines.

For gene-level analysis, transcript counts were aggregated by summing unique counts. For ambiguous counts, the mean across all transcripts per gene was used, assuming transcripts from the same gene multimap to the same genes. PolyASE visualizes allelic ratios and filters genes with high multimapping rates or length differences between haplotypes.

Multimapping rates were calculated for each syntenic group as: $$gene\,ambiguity\,ratio $$$$= (total\,multimapping\,counts) $$$$/(total\,unique + multimapping\,counts) $$. This ratio is identical for all genes within a syntenic group and filters groups where allelic expression cannot be reliably tested for allelic imbalance due to similarity to other genes. Allelic ratios were calculated as: $$allelic\,ratio\,perallele $$$$= unique\,counts\,per\,allele/unique\,counts\,per\,gene $$. For each transcript, the ambiguity ratio was computed as: *transcript ambiguityratio *$$= (multimapping\,counts\,per\,transcript) $$$$/(unique + multimapping\,counts\,per\,transcript) $$. Gene-level transcript ambiguity was calculated as a weighted average: $$ $$$$ gene\,level\,transcript\,ambiguity $$$$ $$$$ = \frac{{\sum\limits_{{i = 0}}^{n} t ranscript\,ambiguity\,ratio_{i} \,x\,total\,transcript\,counts_{i} }}{{gene\,counts}} $$. This metric filters genes unsuitable, due to high isoform similarity, for differential isoform usage testing.

### Statistical testing

All tests used raw unique counts and a likelihood ratio test based on a beta-binomial model. For allelic imbalance within conditions, each allele was tested against the expected count ratio (1/ploidy × total gene counts per replicate). For comparing allelic ratios between conditions, unique counts from all replicates were compared. For differential isoform usage between conditions, unique counts per isoform were compared.

For multi-isoform genes, PolyASE tests whether the dominant isoform was consistent across haplotypes. Within each syntenic group, we identified the most expressed haplotype and designated its top two isoforms as "major" and "minor" references. Isoforms were matched across haplotypes using structural similarity scores based on exon count and lengths (60% weight) and intron lengths (40% weight), with differences under 10 bp considered identical. PolyASE tests the major isoform if present across all haplotypes, otherwise the minor isoform. Pairwise comparisons between the reference and each non-reference haplotype were performed.

All likelihood ratio tests used the betabinom_lr_test() function from isotools v2.0.0 [5, 35]. P-values were corrected for multiple testing using statsmodels.stats.multitest.multipletests() with method = "fdr_bh" [4]. By default, genes where at least one allele had FDR < 0.05 and a mean ratio difference > 0.1 from the expected ratio were considered to have allelic imbalance or significant difference in allelic expression between conditions. For isoform-level analysis, genes where at least one isoform had FDR < 0.05 and mean isoform ratio differences exceeding 0.2 were classified as having differential isoform usage or haplotype-specific differential isoforms.

For visualization, counts per million (CPM) are calculated from total unique counts per sample and plotted using RNApysoforms v1.2.12 [1] to compare isoform usage and allele-specific structures.

### Application to crop datasets

Sources of all genome annotations and assemblies and long-read RNA-seq datasets used can be found in Supplementary Table S1. Organellar genomes were appended to the respective assemblies, and prefixes were added to gene identifiers to distinguish haplotypes.

Annotation transfer with Liftoff was run independently for each chromosome and haplotype with parameters "-copies -a 0.9 -s 0.9" for rice ‘Nipponbare’ and potato ‘Atlantic’. The Syntelogfinder pipeline v1.0.0 was run on the liftoff annotation of rice ‘Nipponbare’ and potato ‘Atlantic’ and the original annotations of potato ‘Atlantic’, sweet potato ‘Beauregard’ [52], wheat ‘Aikang 58’ [28], strawberry 'Royal Royce' [22], rapeseed ‘Darmor-bzh‘ [44], with reference genome and ploidy of the respective organism.

For *Oryza sativa ssp. japonica ‘*Nipponbare’, a publicly available ONT long-read RNA-seq dataset of rice tillers at four different developmental stages (L, E, M, S) [29] with three biological replicates was obtained. We compared leaf (L) and early tillering (E) stages. For autotetraploid potato ‘Atlantic’ [24], a publicly available ONT long-read RNA-seq dataset with five biological replicates from leaf and tuber tissues was obtained. For the allotetraploid *Brassica napus*, a PacBio Iso-Seq dataset consisting of three biological replicates from leaf tissue grown under control and heat conditions from the cultivar ‘Sentry Summer Rape’ was obtained [3]. Finally, for the allooctoploid strawberry ‘Royal Royce’, a publicly available PacBio Iso-Seq dataset from fruit tissue at early (White, SmallGreen, LargeGreen) and late (Turning, Red, Overripe) maturation stages was used [22].

Long-read RNA-seq samples were processed with the longrnaseq pipeline v1.0.0 using Nextflow v25.04.3 and flag "–technology ONT" for rice and potato and flag “–technology PacBio” for rapeseed, wheat and strawberry. For wheat in addition the flag ‘–large_genome’ was used. Downsampling rates performed to keep BAM file sizes manageable for visualization were 0.99 for potato and rapeseed and 0.1 for rice and strawberry.

For gene-level allelic expression analysis with PolyASE in rice, potato, rapeseed and strawberry, the following settings were used. Only syntenic groups where all replicates had > 20 unique reads were retained and single-copy syntelogs present on all haplotypes were selected. For testing allelic differences within conditions, genes were further filtered to retain only those with the same number of isoforms across haplotypes, < 1/ploidy multimapping ratio, and equal CDS lengths.

For isoform-level analysis, only alleles with a minimum expression of 10 EM counts reported by oarfish in all replicates were retained. Genes with gene-level transcript ambiguity exceeding 0.25 were excluded from the analysis. For testing differences in major isoforms between haplotypes, a minimum similarity of 0.9 was required to match transcripts between haplotypes. For statistical tests default cutoffs (FDR < 0.05 and min ratio difference > 0.1) were used.

### Use cases

We tested the Syntelogfinder approach on six crops with varying ploidy levels: diploid rice, tetraploid potato and rapeseed, hexaploid sweet potato and wheat, and octoploid strawberry. For rice and potato, for which ONT long-read RNA-seq datasets were available, and for rapeseed and strawberry, for which PacBio long-read RNA-seq datasets were available we tested the end-to-end framework for analyzing allele-specific gene and isoform expression. For rice, we used samples from leaf and early tillering developmental stages, for potato, we used samples from tubers and leaf tissue, for rapeseed we used samples from leaves grown under control and heat conditions and for strawberry, we used samples from different stages of fruit ripening. Runtime and peak memory usage for the different datasets can be found in Supplementary Table S5.

### Identification of syntenic genes

**Fig. 2:**
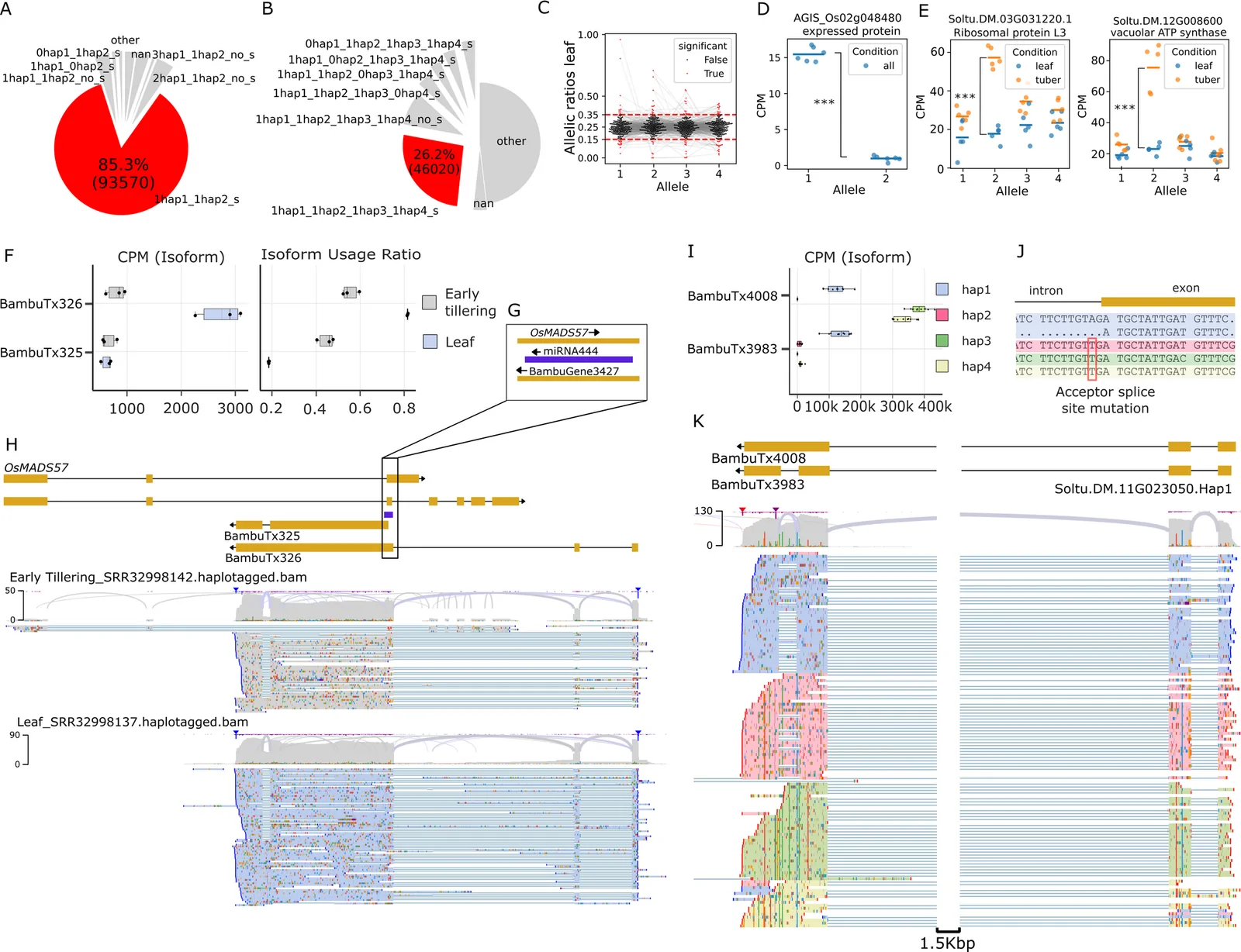
Analysis results of end-to-end framework for allele-specific and isoform expression analysis. Pie charts showing the proportion of genes assigned to syntenic groups for **A** rice ‘Nipponbare’ and **B** potato ‘Atlantic’. Red indicates single-copy syntelogs, which are present on each haplotype with no gene duplications within any haplotype. Other categories follow the naming convention: [copy number]_hap[haplotype ID]_[synteny status], where '_s' indicates syntenic gene order and '_no_s' indicates non-syntenic genes. **C** Swarm plot of allelic ratios for syntelogs in potato leaf samples. Alleles with FDR < 0.05 and mean allelic ratio > 0.35 or < 0.15 are shown in red. Alleles per syntelog are connected with light-gray lines **D** Scatter plot of counts per million (CPMs) for rice genes with allelic imbalance. **E** Scatter plots of CPMs for potato genes with differences in allelic expression between tuber and leaf tissue. Horizontal lines indicate the mean of biological replicates. **F** Box plot of isoform CPMs and isoform usage ratios for the newly annotated rice gene BambuGene3427 with putative differential isoform expression. **G** Overlap of the newly identified gene BambuGene3427 with miRNA444 and *OsMADS57*. **H** Screenshot of JBrowse2 showing alignment of long-read RNA-seq from L and E samples and structures of newly identified isoforms. **I** Box plot of CPMs for isoforms with different major isoform expression across haplotypes in potato. **J** Multiple sequence alignment of haplotypes at the splice site with acceptor splice site mutation in haplotypes 2, 3, and 4. **K** Screenshot of JBrowse2 showing alignment of long-read RNA-seq sorted and colored by haplotype

We tested the framework for identification of syntenic relationships on phased assemblies of diploid rice ‘Nipponbare’ [45], autotetraploid potato ‘Atlantic’ [24], autohexaploid sweet potato ‘Beauregard’ [52], allohexaploid wheat ‘Aikang 58’ [28], allooctoploid strawberry ‘Royal Royce’ [22] and allotetraploid rapeseed ‘Darmor-bzh’ [44]. Across all assemblies, a large portion of genes were identified as single-copy syntelogs on each haplotype/subgenome: 85% in diploid rice (Fig. 2A), 26% in tetraploid potato (Fig. 2B), 47% in hexaploid sweet potato, 44% in hexaploid wheat, 35% in octoploid strawberry and 49% in tetraploid rapeseed (Supplementary Fig. S1A–D).

For potato ‘Atlantic’, the original gene annotations of syntelogs exhibit large length differences due to inconsistencies in UTR annotations (Supplementary Fig. S2 C), that introduces a bias in transcript quantification [40]. To resolve this bias, either the coding sequences (CDS) can be used as a mapping reference, or gene annotations can be unified between haplotypes using Liftoff. The majority of syntelogs had more than 20% length differences between the longest and the shortest gene model on different haplotypes (Supplementary Fig. S2C). We mitigated the bias by lifting over the reference double-monoploid’s Unitato gene models [53] to the individual haplotypes of the phased assembly using Liftoff. Thereafter, most of the syntelogs showed no length difference between haplotypes (Supplementary Fig. S2D). We observed similar annotation length bias in the original annotations of wheat and sweet potato (Supplementary Fig. S2A, B). In the diploid rice gene annotation, which was obtained by lifting over gene annotations from a haploid ‘Nipponbare’ assembly [45], syntelogs showed no length difference between haplotypes (Supplementary Fig. S2E).

### Long-read RNA-seq QC and identification of novel transcripts

Further analysis was performed in rice, potato, rapeseed and strawberry for which long-read RNA-seq datasets were available. Quality metrics of raw reads were consistent across samples (Supplementary Table S2). Error rates averaged 5.5%, 7.2%, 14.5%, 0.4% and mapping rates averaged 98%, 82%, 92% and 100% for rice, potato, rapeseed and strawberry respectively. Principal component analysis showed clear separation of samples by tissue in potato, developmental stage in rice, growing condition in rapeseed and fruit maturation in strawberry (Supplementary Fig. S3A–D).

Bambu identified 1521, 3363, 7598 and 286 novel genes; and 3278, 7878, 18312 and 5275 novel transcripts in the rice, potato, rapeseed and strawberry datasets, respectively. By lifting over these novel transcripts to other haplotypes, 1326 (572) additional transcripts (genes) were added to rice, 8627 (3446) to potato, 6905 (4237) to rapeseed, and 3627 (229) to strawberry annotation. SQANTI-reads analysis confirmed that reads of all samples exhibited comparable proportions of transcript structures classifications. Most reads were classified as ‘full-splice-matches’ (rice 56–70%, potato 50–70%, Supplementary Fig. S4A, B). Most of the remaining reads were assigned as ‘incomplete-splice-matches’ and ‘novel-in-catalog’.

## Allelic expression quantification

Transcript-level counts were aggregated at the gene-level to assess allelic imbalance between haplotypes and conditions, while transcript counts were used to evaluate differential isoform usage and structural variation between alleles.

### Gene-level *Cis*-regulatory differences

To investigate *cis*-regulatory variation, we compared expression levels of syntelogs across haplotypes within the same condition or tissue.

In rice ‘Nipponbare’, we tested for imbalance in allelic expression in the leaf developmental stage. The highly inbred rice cultivar exhibited minimal allelic variation, with only 97 of 46,599 syntelogs containing allelic variants (Supplementary Fig. S5). After filtering, only two genes (AGIS_Os12g030940 and AGIS_Os02g048480) were tested, both showing haplotype 1-dominant expression (Fig. 2D). For AGIS_Os02g048480, read alignment inspection revealed expression exclusively from haplotype 1 (Supplementary Fig. S6), suggesting transcriptional silencing of haplotype 2, which contains two 3' UTR SNPs and a one-base-pair deletion. No promoter variation was detected within 4 kb upstream of the transcription start site, suggesting epigenetic regulation (e.g. DNA methylation) or distal regulatory control.

In ‘Atlantic’ potato, which is highly heterozygous, we tested for imbalanced expression between haplotypes within the leaf samples. After filtering for length equality, SNP presence and expression level, 211 out of 11,505 syntenic gene groups were tested, with 59 showing significant allelic imbalance at FDR < 0.05 and minimum mean allelic ratio difference > 0.1 (Supplementary Table S3). All haplotypes showed similar proportions of balanced and unbalanced alleles (Fig. 2C). In strawberry 'Royal Royce', we tested for unequal expression between the four subgenomes and 51 from 236 tested syntenic genes showed an imbalanced expression (Supplementary Table S6). These results demonstrate how our long-read RNA-seq framework facilitates identification of alleles with imbalanced expression.

### Gene-level *Trans*-regulatory differences

Since *cis*-regulatory elements associated with each haplotype remain fixed, condition-dependent changes in allelic expression can be caused by *trans*-regulatory factors that vary in abundance across experimental conditions. In rice ‘Nipponbare’, none of the tested syntelogs showed differential allelic expression between the leaf and early tillering development stage.

To detect trans-regulatory variation in potato ‘Atlantic’ and rapeseed ‘Sentry Summer Rape’, we tested for differential allelic expression between leaf and tuber tissues and between control and heat-stress conditions, respectively. Of the 342 syntelogs tested in potato, 165 showed significant differences in the relative expression of at least one allele between tissues (FDR < 0.05 and minimum mean ratio difference > 0.1; Supplementary Table S4). For example, two genes exhibited tissue-specific allelic imbalance, with haplotype 2 showing higher expression in tubers for both a ribosomal protein L3 gene (ATL_Soltu.DM.12G008600) and a vacuolar ATP synthase gene (ATL_Soltu.DM.03G031220) (Fig. 2E), suggesting that the promoters of these haplotype 2 alleles may contain tuber-specific regulatory elements. In rapeseed, 141 of the 7,411 genes tested showed differential allelic expression between conditions (Supplementary Table S7).

### Differential isoform usage between conditions

We tested whether the relative expression of gene isoforms changes between conditions, which can indicate differences in isoform function or condition-specific splicing regulation.

In rice, we identified differential isoform usage of a newly annotated gene (BambuGene3427) with two isoforms (BambuTx326 and BambuTx325). Isoform BambuTx325 showed consistent expression levels between developmental stages, whereas BambuTx326, with a retained intron, is more expressed in the leaf stage (Fig. 2F). This Bambu-identified gene is predicted to be a non-coding RNA, partially overlapping the tillering regulation gene *OsMADS57* (Fig. 2H) on the opposite DNA strand. The overlapping region includes a sequence with 142 bp overlap to rice pre-miR444 (Fig. 2G) suggesting this gene encodes miR444 (Supplementary Fig. S7). The role of miR444 in suppressing *OsMADS57* and controlling tillering has been experimentally validated in independent studies [21], providing functional support for this annotation. Notably, *OsMADS57* and the pre-miRNA are present only on haplotype 1. This example demonstrates that our pipeline can identify novel genes and transcripts with differential isoform usage that potentially have functional implications.

### Differences in isoform structure between haplotypes

Long-read RNA-seq allows haplotype-resolved measurement of allelic isoform expression and determining whether the predominant isoform expressed from each haplotype shares the same exon–intron structure. The PolyASE package identifies matching isoforms between haplotypes by comparing exon–intron architectures. In potato, we identified 3 out of 228 groups of single-copy syntelogs present once on each haplotype, where the major expressed isoform differed structurally between haplotypes.

One such gene is Soltu.DM.11G023050, for which we identified three isoforms (two of them novel ones) showing differential isoform usage (Fig. 2I). On haplotype 1, BambuTx4008 and BambuTx3983 were equally expressed, whereas on haplotypes 2, 3, and 4, almost exclusively BambuTx4008 was expressed. This isoform contains a retained intron compared to BambuTx3983 (Fig. 2K). Multiple sequence alignment revealed that haplotypes 2, 3, and 4 share a mutation in the splice acceptor site (AG → TG), directly observed in the long-read sequencing data across all three haplotypes, which is consistent with the observed intron retention (Fig. 2J). This example shows that our framework can be effectively used to identify haplotype-specific variations that alter splicing patterns.

## Discussion

We present a computational pipeline specifically designed for allele-specific gene and isoform expression analysis in polyploid organisms using long-read RNA-seq data. Our framework works with datasets of any ploidy level, thus it can be applied to any species with a phased reference genome.

Although pipelines similar to parts of our framework exist, they do not perform haplotype-level quantification of transcripts and cannot handle polyploid genome sequences. nf-nanoseq [23] performs quantification of known and novel isoforms from ONT long-read RNA-seq, though unlike our framework, it does not process PacBio long-read RNA-seq or include SQANTI3-based quality assessment. TappAS [11] conducts differential isoform usage analysis of novel isoforms using short- or long-read RNA-seq, but is not suitable for phased polyploid genomes.

Several challenges remain for the analysis of allelic expression in polyploids. Independent novel transcript discovery on each haplotype creates annotation inconsistencies that complicate comparative isoform analysis. Our implementation using Liftoff partially addresses this issue, but it also has limitations such as the propagation of wrong gene models, increase in similar isoforms and masking of haplotype-specific isoforms. Complete resolution would require improved annotation standardization across haplotypes. Furthermore, interpreting the causes of allelic expression differences is challenging, as the mechanisms of transcriptional regulation are still poorly understood [55], thus requiring confirmatory wet lab experiments to identify causal regulatory differences.

Pipeline validation and benchmarking were challenging because publicly available FAIR-compliant long-read RNA-seq datasets with several biological replicates, paired with well-annotated phased genomes, remain scarce. However, we expect that with higher adoption, long reads will replace short-read RNA-seq for haplotype-level expression analysis in polyploids. Although optimal depth will depend on ploidy level and allelic complexity, we recommend ~ 100 × coverage per allele at the gene level, achievable with, for example, approximately 3 PacBio Kinnex SMRT cells in a tetraploid potato experiment with 8 biological replicates.

In future releases, we plan to expand the framework to handle polyploid pangenome graphs [50] with the aim of supporting comparison of allele expression across cultivars, enabling analysis of samples without haplotype information [47] and identifying new breeding targets through pan-transcriptomic analysis. Enhanced statistical methods could address genes with high ambiguity and copy number variation. Furthermore, allele-specific RNA modification analysis with direct RNA-seq, such as m6A detection, would be a promising future feature. In addition, ongoing advancements in sequencing technologies and analytical tools are likely to improve novel isoform discovery and quantification using long-read RNA-seq.

## Availability and requirements

Project name: LongPolyASE.

Project home page: https://polyase.readthedocs.io/en/latest/index.html

Operating system(s): Platform independent.

Programming language: Groovy, Python.

Other requirements: Nextflow, conda/singularity.

License: MIT License.

## Any restrictions to use by non-academics: -

### Runtime metrics

Minimum recommended hardware: 24 CPU cores, 256 GB RAM, 400 GB storage, and Singularity [31], Docker or Conda. Runtimes and peak memory usage can be found in Supplementary Table S5.

## Supplementary Information

Additional file 1: Table S1: Availability of reference files and sample information of long-read RNA-seq samples. Table S2: Mapping statistics of long-read RNA-seq of rice ‘Nipponbare’ and potato ‘Atlantic’. Table S3: Results of allele-specific expression analysis within control conditions for potato ‘Atlantic’. Table S4: Results of allele-specific expression analysis between tuber and leaf tissue for potato ‘Atlantic’. Table S5: Runtime and peak memory for datasets of different ploidy. Table S6: Results of allele-specific expression analysis within early fruit maturation for strawberry 'Royal Royce'. Table S7: Results of allele-specific expression analysis between control and heat conditions for rapeseed ‘Sentry Summer Rape’.

Additional file 2: Figure S1: Pie charts of syntenic groups of genes. Figure S2: Length differences between syntelogs. Figure S3: PCA plots of transcript counts per sample. Figure S4: SQANTI-reads categories per sample. Figure S5: Barplot of number of syntelogs per identity category for rice. Figure S6: Read alignments to AGIS_Os02g048480 haplotype 2 in rice. Figure S7: Overlap of BambuTx325 and BambuTx326 with pre-miRNA Osmir444d.

## Data Availability

Liftoff gene annotations, including novel genes and isoforms, for potato ‘Atlantic’ and rice ‘Nipponbare’ used in this study, are available on Zenodo (https://doi.org/10.5281/zenodo.17590759). In addition, test data is provided on Zenodo for each component of the framework and data to run the complete PolyASE workflow for potato ‘Atlantic’ samples. The long-read RNA-seq is publicly available at NCBI SRA with the following identifiers: S. tuberosum ‘Atlantic’ leaf and tuber (SRR14993892-SRR14993895, SRR14996168, SRR14995031-SRR14995034, SRR14995933), O. sativa ‘Nipponbare’ tiller axillary buds (SRR32998137 and SRR32998141-SRR32998146), Brassica napus ‘Sentry Summer Rape’ leaves (SRR32430472-SRR32430477), Fragaria × ananassa (‘Royal Royce’) fruit tissue (SRR16002676-SRR16002681) and Triticum aestivum ‘AK58’ (SRR33004955-SRR33004958) leaf and stem. The longrnaseq pipeline is available at https://github.com/NIB-SI/longrnaseq (doi: https://doi.org/10.5281/zenodo.17631222). The Syntelogfinder pipeline is available at https://github.com/NIB-SI/syntelogfinder (doi: https://doi.org/10.5281/zenodo.17627950). The PolyASE Python package is available at https://pypi.org/project/polyase/ (doi: https://doi.org/10.5281/zenodo.17631236). A Docker image is available at https://hub.docker.com/r/nadjano/polyase (tag: 1.3.6). Documentation and tutorials are available at https://polyase.readthedocs.io/en/latest/index.html.

## Abbreviations

ASE: Allele-Specific Expression
BAM: Binary Sequence Alignment Map format
CDS: Coding sequence
CPM: Counts per million
FDR: False discovery rate
Hap: Haplotype
miRNA: MicroRNA
ONT: Oxford Nanopore Technologies
pre-miRNA: Precursor microRNA
QC: Quality control
RNA-seq: RNA sequencing
SNP: Single Nucleotide Polymorphism
UTR: Untranslated region

## References

[1] Aguzzoli Heberle B, Page ML, Gustavsson EK, Ryten M, Ebbert MTW. RNApysoforms: fast rendering interactive visualization of RNA isoform structure and expression in Python. Bioinformatics Adv. 2024;5(1):vbaf057. 10.1093/bioadv/vbaf057. PMC11964586 40177266

[2] Altschul SF, Gish W, Miller W, Myers EW, Lipman DJ. Basic local alignment search tool. J Mol Biol. 1990;215(3):403–10. 10.1016/S0022-2836(05)80360-2. 2231712

[3] Bailey RE, Adams KL. Transcript isoforms and alternative splicing in polyploid *Brassica napus* under heat and cold stress. Ann Bot. 2026;137(1):181–95. 10.1093/aob/mcaf220. PMC12784076 41081603

[4] Benjamini Y, Hochberg Y. Controlling the false discovery rate: a practical and powerful approach to multiple testing. J R Stat Soc Ser B Stat Methodol. 1995;57(1):289–300. 10.1111/j.2517-6161.1995.tb02031.x.

[5] Bi Y, Lankenau TL, Lienhard M, Herwig R. IsoTools 2.0: software for comprehensive analysis of long-read transcriptome sequencing data. J Mol Biol. 2025;437(15):169049. 10.1016/j.jmb.2025.169049. 40021050

[6] Chen Y, Davidson NM, Wan YK, Yao F, Su Y, Gamaarachchi H, et al. A systematic benchmark of Nanopore long-read RNA sequencing for transcript-level analysis in human cell lines. Nat Methods. 2025;22(4):801–12. 10.1038/s41592-025-02623-4. PMC11978509 40082608

[7] Chen Y, Sim A, Wan YK, Yeo K, Lee JJX, Ling MH, et al. Context-aware transcript quantification from long-read RNA-seq data with Bambu. Nat Methods. 2023;20(8):1187–95. 10.1038/s41592-023-01908-w. PMC10448944 37308696

[8] Chin C-S, Peluso P, Sedlazeck FJ, Nattestad M, Concepcion GT, Clum A, et al. Phased diploid genome assembly with single-molecule real-time sequencing. Nat Methods. 2016;13(12):1050–4. 10.1038/nmeth.4035. PMC5503144 27749838

[9] Cleary S, Seoighe C. Perspectives on allele-specific expression. Annu Rev Biomed Data Sci. 2021;4(1):101–22. 10.1146/annurev-biodatasci-021621-122219. 34465174

[10] Danecek P, Bonfield JK, Liddle J, Marshall J, Ohan V, Pollard MO, et al. Twelve years of SAMtools and BCFtools. Gigascience. 2021;10(2):giab008. 10.1093/gigascience/giab008. PMC7931819 33590861

[11] De La Fuente L, Arzalluz-Luque Á, Tardáguila M, Del Risco H, Martí C, Tarazona S, et al. TappAS: a comprehensive computational framework for the analysis of the functional impact of differential splicing. Genome Biol. 2020;21(1):119. 10.1186/s13059-020-02028-w. PMC7236505 32423416

[12] Degner JF, Marioni JC, Pai AA, Pickrell JK, Nkadori E, Gilad Y, et al. Effect of read-mapping biases on detecting allele-specific expression from RNA-sequencing data. Bioinformatics. 2009;25(24):3207–12. 10.1093/bioinformatics/btp579. PMC2788925 19808877

[13] Di Tommaso P, Chatzou M, Floden EW, Barja PP, Palumbo E, Notredame C. Nextflow enables reproducible computational workflows. Nat Biotechnol. 2017;35(4):316–9. 10.1038/nbt.3820. 28398311

[14] Diesh C, Stevens GJ, Xie P, De Jesus Martinez T, Hershberg EA, Leung A, et al. JBrowse 2: a modular genome browser with views of synteny and structural variation. Genome Biol. 2023;24(1):74. 10.1186/s13059-023-02914-z. PMC10108523 37069644

[15] Dong L, Wang J, Wang G. BYASE: a Python library for estimating gene and isoform level allele-specific expression. Bioinformatics. 2020;36(19):4955–6. 10.1093/bioinformatics/btaa636. 32678892

[16] Emms DM, Kelly S. OrthoFinder: phylogenetic orthology inference for comparative genomics. Genome Biol. 2019;20(1):238. 10.1186/s13059-019-1832-y. PMC6857279 31727128

[17] Ewels PA, Peltzer A, Fillinger S, Patel H, Alneberg J, Wilm A, et al. The nf-core framework for community-curated bioinformatics pipelines. Nat Biotechnol. 2020;38(3):276–8. 10.1038/s41587-020-0439-x. 32055031

[18] Ewels P, Magnusson M, Lundin S, Käller M. MultiQC: summarize analysis results for multiple tools and samples in a single report. Bioinformatics. 2016;32(19):3047–8. 10.1093/bioinformatics/btw354. PMC5039924 27312411

[19] Glinos DA, Garborcauskas G, Hoffman P, Ehsan N, Jiang L, Gokden A, et al. Transcriptome variation in human tissues revealed by long-read sequencing. Nature. 2022;608(7922):353–9. 10.1038/s41586-022-05035-y. PMC10337767 35922509

[20] Godec T, Beier S, Rodriguez-Granados NY, Sasidharan R, Abdelhakim L, Teige M, et al. Haplotype-resolved genome assembly of the tetraploid potato cultivar Désirée. Sci Data. 2025;12(1):1044. 10.1038/s41597-025-05372-3. PMC12181383 40541992

[21] Guo S, Xu Y, Liu H, Mao Z, Zhang C, Ma Y, et al. The interaction between OsMADS57 and OsTB1 modulates rice tillering via DWARF14. Nat Commun. 2013;4(1):1566. 10.1038/ncomms2542. PMC3615354 23463009

[22] Hardigan MA, Feldmann MJ, Pincot DDA, Famula RA, Vachev MV, Madera MA, et al. Blueprint for phasing and assembling the genomes of heterozygous polyploids: application to the octoploid genome of strawberry. Genomics. 2021. 10.1101/2021.11.03.467115.

[23] Patel H, Kei Wan Y, Wratten L, Sawyer C, Ying C, Hakkaart C, Bot NC, Alneberg J, Ekushele, Ewels P, Malladi V, Garcia MU, Schreyer D, Menden K. nf-core/nanoseq: Nf-core/nanoseq v3.1.0—NObelium Guppy (Version 3.1.0) [Computer software]. Zenodo; 2023. 10.5281/ZENODO.7716033

[24] Hoopes G, Meng X, Hamilton JP, Achakkagari SR, Guesdes DAF, F., Bolger, M. E., Coombs, J. J., Esselink, D., Kaiser, N. R., Kodde, L., Kyriakidou, M., Lavrijssen, B., Van Lieshout, N., Shereda, R., Tuttle, H. K., Vaillancourt, B., Wood, J. C., De Boer, J. M., Bornowski, N., et al. Phased, chromosome-scale genome assemblies of tetraploid potato reveal a complex genome, transcriptome, and predicted proteome landscape underpinning genetic diversity. Mol Plant. 2022;15(3):520–36. 10.1016/j.molp.2022.01.003. 35026436

[25] Huang N, Human Pangenome Reference Consortium, Li H. SNP calling, haplotype phasing and allele-specific analysis with long RNA-seq reads. Bioinformatics. 2025. 10.1101/2025.05.26.656191 PMC13041723 41912802

[26] Huynen MA, Bork P. Measuring genome evolution. Proc Natl Acad Sci U S A. 1998;95(11):5849–56. 10.1073/pnas.95.11.5849. PMC34486 9600883

[27] Dainat J, Cannoodt R, Soares A, Ruano DG, Hereñú D, Murray DK, Davis E, Ugrin I, Crouch K, Soler L, Agostinho N, Pascal-git, Zollman Z, Tayyrov. NBISweden/AGAT: AGAT v1.5.1 (Version v1.5.1) [Computer software]. Zenodo. 2025. 10.5281/ZENODO.3552717

[28] Jia J, Zhao G, Li D, Wang K, Kong C, Deng P, et al. Genome resources for the elite bread wheat cultivar Aikang 58 and mining of elite homeologous haplotypes for accelerating wheat improvement. Mol Plant. 2023;16(12):1893–910. 10.1016/j.molp.2023.10.015. 37897037

[29] Jiangxi Agricultural University. Iso-seq of upland rice (OXFORD_NANOPORE (PromethION) No. PRJNA1247236). Sequence Read Archive (SRA). 2025.

[30] Keil N, Monzó C, McIntyre L, Conesa A. Quality assessment of long read data in multisample lrRNA-seq experiments using SQANTI-reads. Genome Res. 2025;35(4):987–98. 10.1101/gr.280021.124. PMC12047275 40032587

[31] Kurtzer GM, Sochat V, Bauer MW. Singularity: scientific containers for mobility of compute. PLoS ONE. 2017;12(5):e0177459. 10.1371/journal.pone.0177459. PMC5426675 28494014

[32] Langer BE, Amaral A, Baudement M-O, Bonath F, Charles M, Chitneedi PK, et al. Empowering bioinformatics communities with Nextflow and nf-core. Genome Biol. 2025;26(1):228. 10.1186/s13059-025-03673-9. PMC12309086 40731283

[33] Li H. Minimap2: pairwise alignment for nucleotide sequences. Bioinformatics. 2018;34(18):3094–100. 10.1093/bioinformatics/bty191. PMC6137996 29750242

[34] Li H. New strategies to improve minimap2 alignment accuracy. Bioinformatics. 2021;37(23):4572–4. 10.1093/bioinformatics/btab705. PMC8652018 34623391

[35] Lienhard M, Van Den Beucken T, Timmermann B, Hochradel M, Börno S, Caiment F, et al. IsoTools: a flexible workflow for long-read transcriptome sequencing analysis. Bioinformatics. 2023;39(6):btad364. 10.1093/bioinformatics/btad364. PMC10287928 37267159

[36] Lovell JT, Sreedasyam A, Schranz ME, Wilson M, Carlson JW, Harkess A, et al. GENESPACE tracks regions of interest and gene copy number variation across multiple genomes. Elife. 2022;11:e78526. 10.7554/eLife.78526. PMC9462846 36083267

[37] Mattevi S, Mazzarotto F, Martini P. Allele-specific expression analysis: pipelines, applications, challenges, and unmet needs. Comput Biol Med. 2025;196:110890. 10.1016/j.compbiomed.2025.110890. 40759060

[38] Metzger BPH, Duveau F, Yuan DC, Tryban S, Yang B, Wittkopp PJ. Contrasting frequencies and effects of cis—and trans -regulatory mutations affecting gene expression. Mol Biol Evol. 2016;33(5):1131–46. 10.1093/molbev/msw011. PMC4909133 26782996

[39] Monzó C, Liu T, Conesa A. Transcriptomics in the era of long-read sequencing. Nat Rev Genet. 2025;26(10):681–701. 10.1038/s41576-025-00828-z. 40155769

[40] Nolte N, Petek M, Angulo Lara P, Mulroney L, Nicassio F, Marroni F, et al. The promise of long-read RNA-seq: reducing bias in analyses of allele imbalance. Genetics. 2025. 10.1101/2025.10.14.682301. PMC13434171 42553517

[41] Pardo-Palacios FJ, Arzalluz-Luque A, Kondratova L, Salguero P, Mestre-Tomás J, Amorín R, et al. SQANTI3: curation of long-read transcriptomes for accurate identification of known and novel isoforms. Nat Methods. 2024;21(5):793–7. 10.1038/s41592-024-02229-2. PMC11093726 38509328

[42] Pertea G, Pertea M. GFF utilities: GffRead and GffCompare. F1000Res. 2020;9:304. 10.12688/f1000research.23297.1. PMC7222033 32489650

[43] Quinones-Valdez G, Amoah K, Xiao X. Long-read RNA-seq demarcates cis- and trans-directed alternative RNA splicing. Nat Commun. 2025;16(1):9603. 10.1038/s41467-025-64605-6. PMC12575695 41168189

[44] Rousseau-Gueutin M, Belser C, Da Silva C, Richard G, Istace B, Cruaud C, et al. Long-read assembly of the *Brassica napus* reference genome Darmor-bzh. GigaScience. 2020;9(12):giaa137. 10.1093/gigascience/giaa137. PMC7736779 33319912

[45] Shang L, He W, Wang T, Yang Y, Xu Q, Zhao X, et al. A complete assembly of the rice Nipponbare reference genome. Mol Plant. 2023;16(8):1232–6. 10.1016/j.molp.2023.08.003. 37553831

[46] Shumate A, Salzberg SL. Liftoff: accurate mapping of gene annotations. Bioinformatics. 2021;37(12):1639–43. 10.1093/bioinformatics/btaa1016. PMC8289374 33320174

[47] Sibbesen JA, Eizenga JM, Novak AM, Sirén J, Chang X, Garrison E, et al. Haplotype-aware pantranscriptome analyses using spliced pangenome graphs. Nat Methods. 2023;20(2):239–47. 10.1038/s41592-022-01731-9. 36646895

[48] Sun H, Jiao W-B, Krause K, Campoy JA, Goel M, Folz-Donahue K, et al. Chromosome-scale and haplotype-resolved genome assembly of a tetraploid potato cultivar. Nat Genet. 2022;54(3):342–8. 10.1038/s41588-022-01015-0. PMC8920897 35241824

[49] Tang AD, Felton C, Hrabeta-Robinson E, Volden R, Vollmers C, Brooks AN. Detecting haplotype-specific transcript variation in long reads with FLAIR2. Genome Biol. 2024;25(1):173. 10.1186/s13059-024-03301-y. PMC11218413 38956576

[50] Wang C, Han B. From sugarcane to polyploid crops: graph pangenomes usher in a new era of complex-genome breeding. Plant Commun. 2026;7(5):101852. 10.1016/j.xplc.2026.101852. PMC13174201 41981909

[51] Wang Y, Tang H, DeBarry JD, Tan X, Li J, Wang X, et al. MCScanX: A toolkit for detection and evolutionary analysis of gene synteny and collinearity. Nucleic Acids Res. 2012;40(7):e49. 10.1093/nar/gkr1293. PMC3326336 22217600

[52] Wu S, Sun H, Zhao X, Hamilton JP, Mollinari M, Gesteira GDS, et al. Phased chromosome-level assembly provides insight into the genome architecture of hexaploid sweetpotato. Nat Plants. 2025;11(9):1951–9. 10.1038/s41477-025-02079-6. 40781486

[53] Zagorščak M, Zrimec J, Bleker C, Nolte N, Juteršek M, Ramšak Ž, et al. Evidence-based unification of potato gene models with the UniTato collaborative genome browser. Front Plant Sci. 2024;15:1352253. 10.3389/fpls.2024.1352253. PMC11196761 38919818

[54] Zare Jousheghani Z, Singh NP, Patro R. Oarfish: enhanced probabilistic modeling leads to improved accuracy in long read transcriptome quantification. Bioinformatics. 2025;41(Supplement_1):i304–13. 10.1093/bioinformatics/btaf240. PMC12261437 40662837

[55] Zeitlinger J, Roy S, Ay F, Mathelier A, Medina-Rivera A, Mahony S, et al. Perspective on recent developments and challenges in regulatory and systems genomics. Bioinformatics Adv. 2024;5(1):vbaf106. 10.1093/bioadv/vbaf106. PMC12233095 40626041

